# Thymus-Associated Parathyroid Hormone Has Two Cellular Origins with Distinct Endocrine and Immunological Functions

**DOI:** 10.1371/journal.pgen.1001251

**Published:** 2010-12-23

**Authors:** Zhijie Liu, Alison Farley, Lizhen Chen, Beth J. Kirby, Christopher S. Kovacs, C. Clare Blackburn, Nancy R. Manley

**Affiliations:** 1 Department of Genetics, University of Georgia, Athens, Georgia, United States of America; 2 Medical Research Council Centre for Regenerative Medicine, Institute for Stem Cell Research, School of Biological Sciences, University of Edinburgh, Edinburgh, United Kingdom; 3 Faculty of Medicine-Endocrinology, Memorial University of Newfoundland, St. John's, Newfoundland, Canada; University of Washington, United States of America

## Abstract

In mammals, parathyroid hormone (PTH) is a key regulator of extracellular calcium and inorganic phosphorus homeostasis. Although the parathyroid glands were thought to be the only source of PTH, extra-parathyroid PTH production in the thymus, which shares a common origin with parathyroids during organogenesis, has been proposed to provide an auxiliary source of PTH, resulting in a higher than expected survival rate for aparathyroid *Gcm2* ^−/−^ mutants. However, the developmental ontogeny and cellular identity of these “thymic” PTH–expressing cells is unknown. We found that the lethality of aparathyroid *Gcm2* ^−/−^ mutants was affected by genetic background without relation to serum PTH levels, suggesting a need to reconsider the physiological function of thymic PTH. We identified two sources of extra-parathyroid PTH in wild-type mice. Incomplete separation of the parathyroid and thymus organs during organogenesis resulted in misplaced, isolated parathyroid cells that were often attached to the thymus; this was the major source of thymic PTH in normal mice. Analysis of thymus and parathyroid organogenesis in human embryos showed a broadly similar result, indicating that these results may provide insight into human parathyroid development. In addition, medullary thymic epithelial cells (mTECs) express PTH in a *Gcm2*-independent manner that requires TEC differentiation and is consistent with expression as a self-antigen for negative selection. Genetic or surgical removal of the thymus indicated that thymus-derived PTH in *Gcm2* ^−/−^ mutants did not provide auxiliary endocrine function. Our data show conclusively that the thymus does not serve as an auxiliary source of either serum PTH or parathyroid function. We further show that the normal process of parathyroid organogenesis in both mice and humans leads to the generation of multiple small parathyroid clusters in addition to the main parathyroid glands, that are the likely source of physiologically relevant “thymic PTH.”

## Author Summary

Due to the important role of PTH in the regulation of physiological activities, disorders in PTH production can cause many diseases in humans. Thus it is very important to understand where PTH is produced and how it is regulated. Many people have been found to have ectopic and supernumerary parathyroid glands without clear ontogenesis. In addition, the thymus, which develops together with the parathyroid during embryogenesis, has been proposed to be an auxiliary source of PTH with endocrine function; however, PTH is also a tissue-restricted self-antigen expressed by the thymus. In this paper, we provide insights into the ontogeny and function of thymus-associated PTH. We found that ectopic and supernumerary parathyroid glands originate from the normal developmental process underlying the separation of parathyroid and thymus, resulting in misplaced parathyroids close or attached to thymus. In the thymus, thymic epithelial cells can produce a low level of PTH via a different mechanism than the parathyroid and provide functional data that TEC-derived PTH does not have endocrine function. In summary, our data show that the thymic source of PTH has no endocrine function and, instead, has an expression pattern in the thymus consistent with that of a self-antigen for negative selection.

## Introduction

Mammals have evolved an integrated system consisting of the parathyroid glands, bone, kidney and the intestine, to regulate ionized calcium and inorganic phosphorus homeostasis in the extracellular environment [1]. Circulating ionized Ca^2+^ and inorganic phosphorus are required for a wide range of physiological activities, including neuromuscular excitability, muscle contraction, energy storage, bone mineralization, blood coagulation and cardiovascular functions. Parathyroid hormone (PTH) produced by the parathyroids acts as the key endocrine regulator to modulate the physiological actions in the bone, kidney and the intestine to maintain the homeostasis of ionized calcium and inorganic phosphorus concentrations in the extracellular environment. Failure of calcium and phosphorus homeostasis, which can result from PTH production disorders, causes serious physiological consequences in human [2].

The parathyroid glands were long thought to be the sole source of PTH production and secretion. However, analysis of the aparathyroid *Gcm2* null mouse mutant phenotype identified the thymus, a primary lymphoid organ, as an auxiliary source of circulating PTH in addition to the parathyroids in mice [3], [4]. Thymic PTH was found to come from small clusters of unidentified cells under the thymic capsule in wild-type mice, although the ontogeny of these intrathymic PTH-expressing cells and the regulation of PTH expression in these cells are not clear. In humans, ectopic parathyroid cells have been found in a variety of different locations, most commonly in the thymus [5], which was thought to account for the origin of intrathymic parathyroid adenomas in some patients [6]. However, the significance of thymus-associated PTH for the survival of *Gcm2* mouse mutants was called into question by the phenotype of *Pth* null mutants, which can survive in the complete absence of PTH [7], [8].

Despite their distinct primary functions, the parathyroid and thymus organs have a close relationship during organogenesis, initially developing from two shared parathyroid/thymus primordia originating from the bilateral 3^rd^ pharyngeal pouches [9]. Analysis of mouse mutants has shown that initial formation and early patterning of the thymus and parathyroid domains are controlled by a common regulatory pathway, including Hoxa3, Pax1, Pax9 and Eya1 [10], [11]. Once the organ domains are specified, their differentiation is regulated by two different organ-specific transcription factors, Gcm2 (for parathyroid) and Foxn1 (for thymus) [9]. In humans the bilateral 3^rd^ and 4^th^ pharyngeal pouches are thought to give rise to four parathyroids [12]–[14]; the pair of inferior parathyroid glands develop together with the thymus from the 3^rd^ pharyngeal pouches, while the pair of superior parathyroid glands (not present in mice) develop with the ultimobranchial bodies from the 4^th^ pharyngeal pouches [5]. Accessory parathyroids have also been reported in animals and in humans; although their origins were difficult to determine by histology alone, these structures were proposed to originate either during organogenesis, or to be induced postnatally in response to experimentally or surgically induced hypoparathyroidism [12]–[15]. Furthermore, intrathymic parathyroid adenomas have been hypothesized to originate from “ectopically migrating parathyroid cells” [6].

The original analysis of the *Gcm2* mutant mouse reported that these mice were aparathyroid from embryonic stages [4]. Our subsequent analysis of the role of Gcm2 in parathyroid organogenesis showed that Gcm2 controls the differentiation and survival of parathyroid precursor cells, but is not required to specify the parathyroid domain within the pouch endoderm [3]. Without Gcm2 function, parathyroid precursor cells fail to differentiate and then undergo apopotosis by embryonic day 12, resulting in an aparathyroid phenotype [3], [4]. Mutation of Gcm2 in humans has also been associated with hypoparathyroidism [16], [17]. However, the role of Gcm2 in the development of extra-parathyroid PTH-expressing cells is as yet unknown.

To clarify the ontogenesis, regulation of PTH expression, and physiological role of extra-parathyroid PTH-expressing cells, we studied parathyroid and thymus organogenesis in the mouse. We showed that clusters of ectopic parathyroid cells between the parathyroid and thymus or attached to the thymus resulting from incomplete separation of these two organs during normal organogenesis. Analysis of parathyroid organogenesis in human embryos showed a similar phenomenon. Absence of these misplaced parathyroid cells in the thymus in *Gcm2* ^−/−^ mice caused a significant decrease of thymic PTH expression but still left a low level of thymic PTH expression, which we identified as originating from mTECs expressing PTH in a Gcm2-independent but Foxn1-dependent manner. Our results indicate that mTEC-derived PTH is not secreted into the general circulation and does not function as a backup mechanism of parathyroid glands, but may function as a self-antigen for negative selection. We further show that the lethality associated with *Gcm2* mutation is not related to the presence of thymic PTH or serum PTH levels. Our results also have implications for the molecular mechanism of promiscuous gene expression of tissue-restricted self-antigens in mTECs. Our data also provide an explanation for the origin of ectopic parathyroid adenomas that are often associated with human hyperparathyroidism.

## Results

### Survival of Aparathyroid *Gcm2* ^−/−^ Mice Is Dependent on Genetic Background without Rescue from Hypoparathyroidism

**Figure 1:**
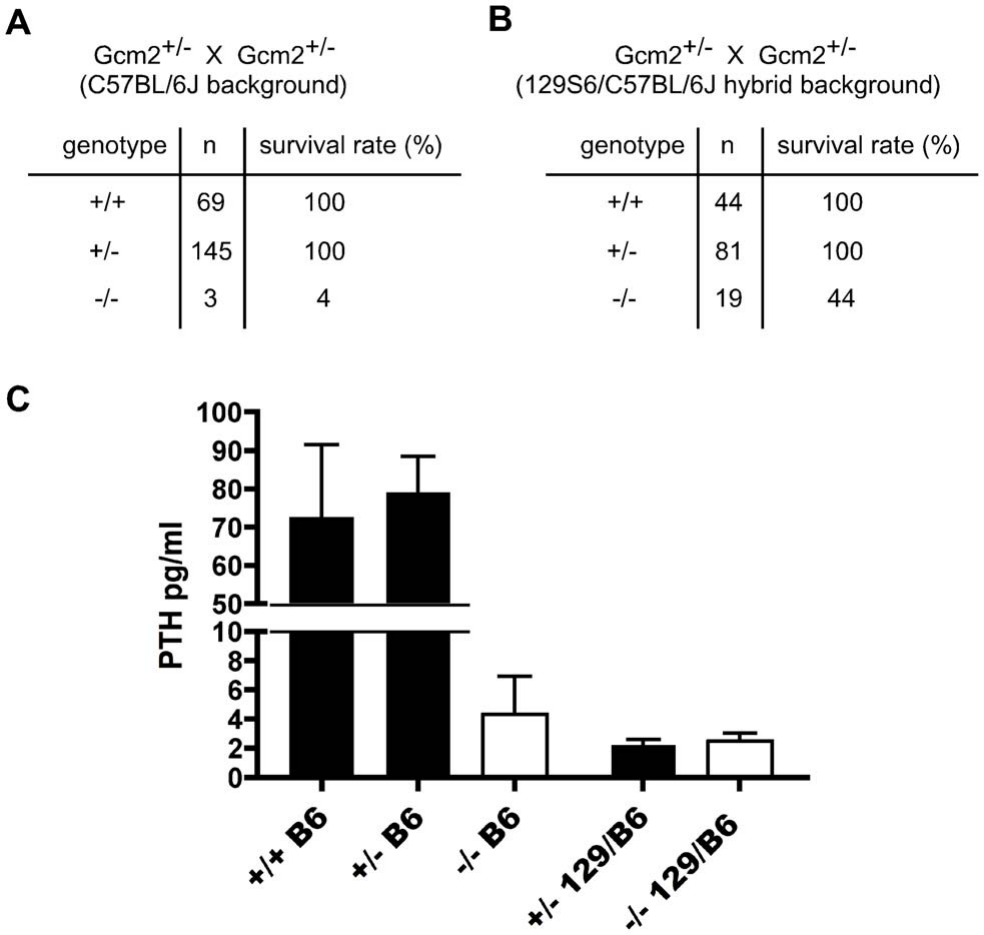
Increased lethality of aparathyroid *Gcm2* ^−/−^ mutants on the C57BL/6J background is unrelated to serum PTH levels. (A) The survival rate of mice born from *Gcm2* ^+/−^ intercrosses on the C57BL/6J genetic background. Most *Gcm2* ^−/−^ mice died at the newborn stage. Mice that survived more than 1 month were counted as survivors. (B) The survival rate of mice born from *Gcm2* ^+/−^ intercrosses on the 129S6-C57BL/6J F1 hybrid background. In A and B, n =  number of surviving adult mice. (C) PTH concentrations of wild-type, *Gcm2* ^+/−^, and *Gcm2* ^−/−^ mice on the C57BL/6J (B6) genetic background (from +/- intercrosses), and of *Gcm2* ^+/−^and *Gcm2* ^−/−^ mice from the hybrid genetic background (from +/− x −/− crosses; most females were +/−). >75% of mutants in both genetic backgrounds were below the level of detection of the assay (these values were reset at the detection limit of 1.6).

**Figure 2:**
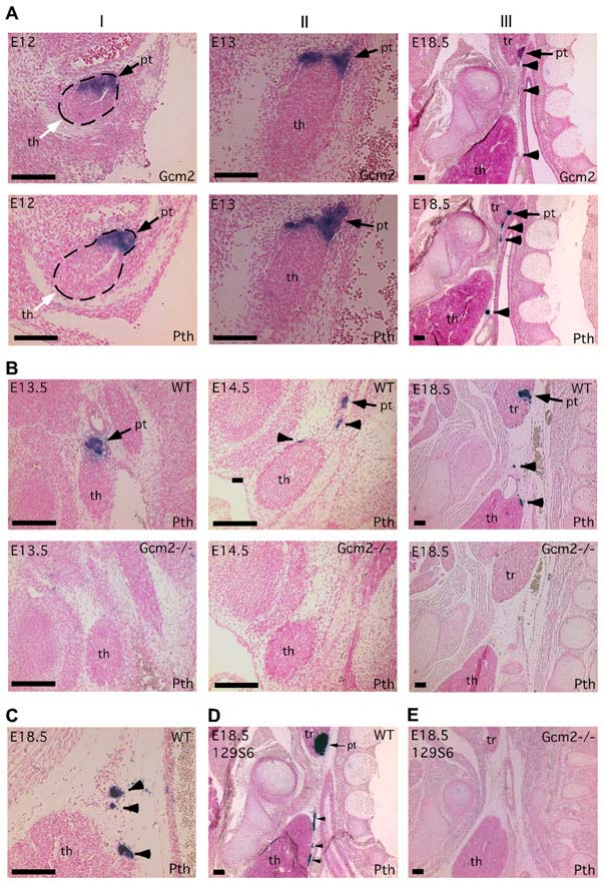
Incomplete separation of parathyroid and thymus organs results in extra-parathyroid PTH production. Paraffin section *in situ* hybridization for *Gcm2* (A) and *Pth* (A–E); sections were cut in the sagittal plane. In all figures, anterior is up, and dorsal is to the right. Ages of embryos are in the upper left corner of each panel. Probes used for *in situ* hybridization are in the lower right. (A) Wild-type embryos at E12 (I), E13 (II) and E18.5 (III) show the separation of parathyroid and thymus organs from the common primordium. The parathyroid/thymus common primordia in panels A–I are outlined. (B) Loss of both parathyroid gland and misplaced parathyroid cells in *Gcm2* ^−/−^ mutants. (C) Location of PTH-expressing cells that were close to or attached to the wild-type thymus. In panels A–C, embryos used were on C57BL/6J genetic background. (D–E) The aparathyroid phenotype caused by *Gcm2* null mutation also happens in the 129S6 genetic background. (D) Section *in situ* hybridization for *Pth* in wild-type E18.5 129S6 embryos shows the primary parathyroid (arrow) and several misplaced parathyroid cells (arrowheads). (E) Analysis of Gcm2 null mutants shows the loss of all parathyroid cells on the 129S6 genetic background. In all panels, black arrows point to the parathyroid. White arrows point to the thymus domain. Arrowheads point to misplaced parathyroid cells. pt, parathyroid; th, thymus; tr, thyroid. Scale bars  = 0.1 mm.

We compared the phenotypes of *Gcm2* ^−/−^ mutants on the C57BL/6J and 129/C57BL/6J F1genetic backgrounds for survival and parathyroid function. We found that *Gcm2* ^−/−^ mutants on a C57BL/6J genetic background had a nearly 100% lethality rate (Figure 1A), compared to 56% on the 129/C57BL/6J F1genetic background (Figure 1B) and to about 30% with additional backcross generations onto 129S6 (Table 1), confirming the original report [4]. Analysis of fetal parathyroid organogenesis in mutants from both genetic backgrounds confirmed our earlier data showing a complete absence of *Pth*-positive parathyroid cells [3] (Figure 2B, 2D and 2E). These data show that the reduced lethality on the 129/C57BL/6J hybrid background is not due to incomplete deletion of the parathyroids.

**Table 1:** Survival rates of thymectomized *Gcm2* ^−/−^ mutants and *Gcm2* ^+/−^ control littermates on the 129S6 background at weaning.

Experimental Group	Total at newborn	Survival at weaning	Survival rate
*Gcm2^+/−^* (no Surgery)	19	19	100%
*Gcm2^−/−^* (no Surgery)	24	17	70.8%
*Gcm2^+/−^* (mock)	16	15	93.8%
*Gcm2^−/−^* (mock)	19	14	73.7%
*Gcm2^+/−^* (thymectomy)	19	15	78.9%
*Gcm2^−/−^* (thymectomy)	10	6	60%

To test whether 129/C57BL/6J hybrid *Gcm2* ^−/−^ mutants had a higher serum PTH concentration than the C57BL/6J *Gcm2* ^−/−^ mutants that failed to survive, we measured serum PTH levels in E18.5 fetal *Gcm2* ^−/−^ mutants with different genetic backgrounds. Most *Gcm2* ^−/−^ mutants on both genetic backgrounds had undetectable serum PTH levels, with only a few individuals of each genetic background showing variable levels above the detection limit (3/23 for 129/C57BL/6J; 3/13 for C57BL/6J; Figure 1C). This dramatic reduction of serum PTH levels in *Gcm2* ^−/−^ mutants is consistent with other reports on a variety of genetic backgrounds [18], [19]. These results show that serum PTH levels in the *Gcm2* ^−/−^ mutants are not affected by genetic background, and that the lethality phenotype observed in *Gcm2* ^−/−^ mutants is not related to serum PTH levels.

Heterozygotes on the 129/C57Bl6 hybrid genetic background also had low or undetectable serum PTH levels. This difference in steady-state PTH levels was not correlated with differences in maternal ionized calcium levels, which were similar in heterozygote and wild-type females from both strains, and parathyroid glands in heterozygotes from the hybrid background were histologically normal (data not shown). Variations in PTH levels have been reported between C3H/HeJ and C57BL/6 mice, including change in PTH levels in response to altering the calcium content of the diet, as well as differences between strains in BMD, calcium absorption, serum calcium, and calcitriol levels [20]. As serum chemistry was normal, this result further supports our observation that serum PTH levels do not correlate with lethality.

### Misplaced Parathyroid Cells Result from Incomplete Parathyroid/Thymus Separation during Organogenesis

To investigate the possible role of thymic PTH in the lethality of *Gcm2* mutants, we designed experiments to determine the ontogenesis of extra-parathyroid PTH-expressing cells. Since the parathyroids and thymus arise from the same embryonic structure, we tracked the process by which the parathyroid and thymus domains resolve into separate primordia in mice using *in situ* hybridization for *Pth* and *Gcm2*. At E12, *Gcm2*/*Pth* expression in the parathyroid/thymus common primordia specifically marked the anterior/dorsal Gcm2-positive parathyroid domain with a clear interface at the posterior/ventral Foxn1-positive thymus domain [3], [21](Figure 2A-I). At E13, the *Gcm2*/*Pth*-positive parathyroid domain had started to separate from the thymus domain, and some parathyroid cells were located outside the major parathyroid domain (Figure 2A-II). At E18.5, small clusters of parathyroid cells were located between the parathyroids and thymus or directly associated with the thymus, in some cases under the developing thymic capsule (Figure 2A-III and 2C). This phenotype was seen in all 11 E16.5-18.5 wild-type embryos on multiple genetic backgrounds (C57BL/6J, 129/C57BL/6J F1 hybrid, or 129S6; Figure 2A–2D), which indicates that this incomplete separation pattern is a common phenomenon in the mouse.

**Figure 3:**
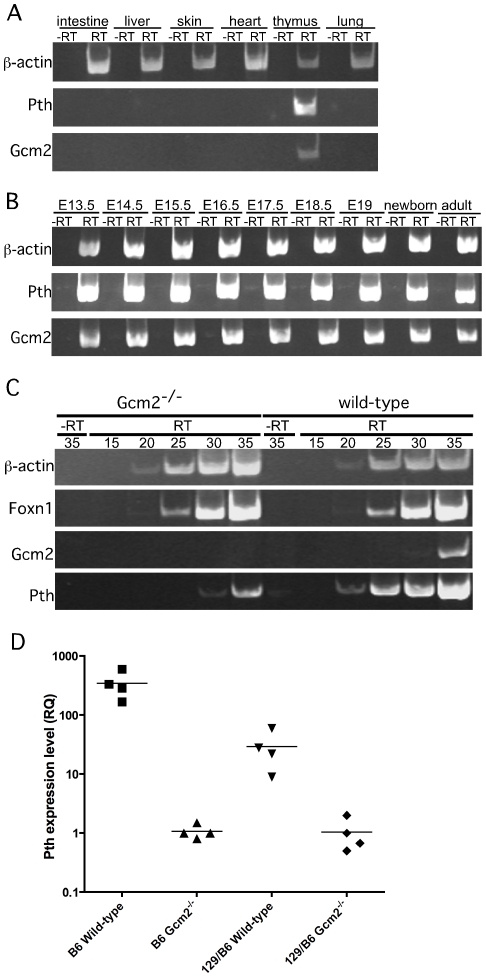
*Pth* expression in misplaced parathyroid cells was co-expressed with *Gcm2* and down-regulated in *Gcm2*-null mutant mice. (A) RT-PCR using cDNA made from different tissues to show the co-expression of *Gcm2* with *Pth* only in thymus, but not in other organs tested. (B) RT-PCR using thymus cDNA from different stages. The expression of *Gcm2* and *Pth* was detected in all stages tested. (C) Semi-quantitative RT-PCR on thymus cDNA from newborn wild-type and *Gcm2* ^−/−^ mutants. PCR amplification cycle numbers are indicated at the top. β-actin was used as a loading control. *Foxn1* was a positive control for TECs. *Gcm2* transcripts were absent and *Pth* transcription levels were much lower in *Gcm2* ^−/−^ mutants. (D) Realtime PCR of *Pth* was performed for total thymus cDNA samples from wild-type and *Gcm2* ^−/−^ mutants on the C57BL/6J or 129S6 (129) and C57BL/6J (B6) F1 hybrid background. In panel D, n = 4. RQ is the relative quantitative expression level of *Pth*.

RT-PCR using cDNA made from total thymus and other organs from wild-type mice confirmed that co-expression of *Gcm2* and *Pth* was detected only in the thymus (Figure 3A). *Gcm2* and *Pth* expression could be detected as early as E13.5 in dissected whole thymus, when the thymus had just separated from the parathyroids, and at all later stages (Figure 3B).

If these misplaced *Gcm2*/*Pth*-positive cells are authentic parathyroid cells, *Gcm2* should regulate their differentiation and survival [3]. As predicted, all misplaced parathyroid cells were ablated in *Gcm2* ^−/−^ mutants (Figure 2B, 2D and 2E). The thymic *Pth* expression level was also greatly reduced relative to wild-type, while the expression of the TEC marker *Foxn1* was not affected (Figure 3C). These data suggest that misplaced parathyroid cells in the thymus are the primary source of thymic PTH in wild-type mice, and that these cells are absent in *Gcm2* ^−/−^ mutants.

### Ectopic Parathyroid Tissue Is Observed in the Human Embryo from Early Week 7

**Figure 4:**
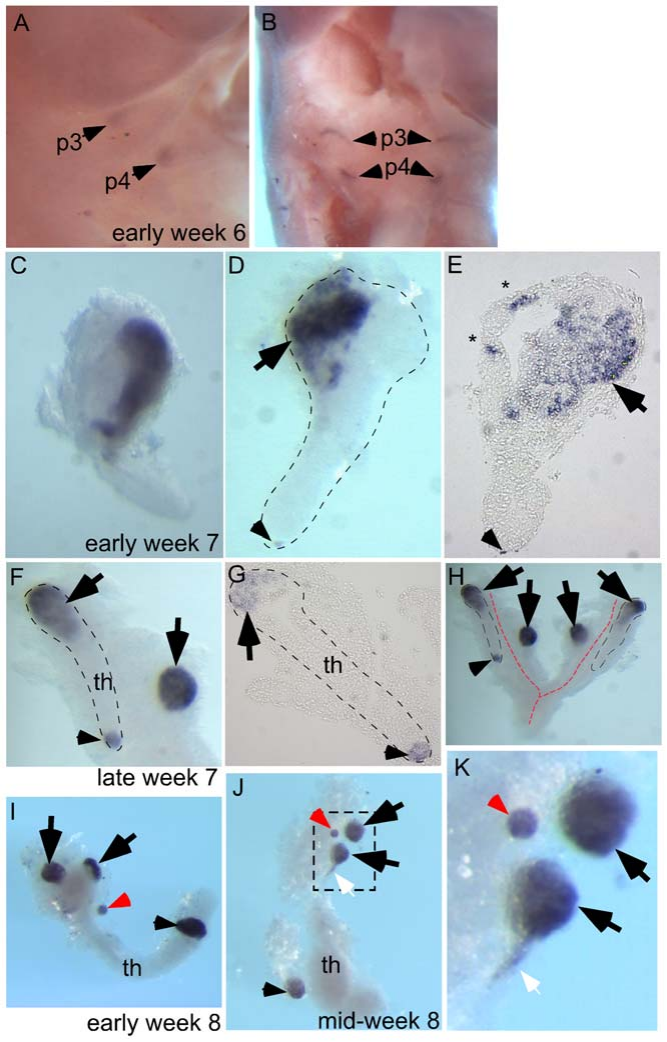
Ectopic parathyroids are present from week 7 in the human embryo. Whole-mount in situ hybridization for *GCM2* (A–D, F, H–K) and whole-mounts embedded in paraffin and sectioned after in situ hybridization (E, G). Ages of embryos or dissected parathyroid/thymus primordia in the lower right corner of A, C, and F apply to the entire row; age in J also applies to K. The entire thymus/parathyroid common primoridum is outlined in D, F, G, and H. In panels D–K, black arrows show presumptive primary parathyroids, small arrowheads indicate *GCM2*-positive clusters at the posterior end of the thymus, and red arrows show probable accessory parathyroids. White arrows in J and K show trailing *GCM2*-positive cells. (A, B) Side (A) and frontal (B) views show *GCM2* expression in the 3^rd^ and 4^th^ pharyngeal pouches in an early week 6 embryo (2/2 embryos). (C–E) Images of whole-mount (C, D) and paraffin sectioned (E, section from D) early to mid week 7 dissected parathyroid/thymus common primordia showing *GCM2* expressing cells present in the anterior (4/4 embryos) and posterior tip (1/4 embryos) of the common primordia. Small ectopic clusters away from the main cluster (*) are also often present by this stage. (F) Image shows separation of parathyroids from the common primordia is occurring by late week 7 (right arrow; 1/1 embryo; the posterior parathyroid was present in only one of the two common primordia in this embryo). (G) Paraffin section of whole mount shown in F. (H) Whole mount showing bilateral primordia (outlined) and carotid artery complex (red dashed line), with four main parathyroids and one small parathyroid cluster on the posterior tip of one thymic lobe. (I, J) Thymic lobes and surrounding parathyroids from one side each of two separate early week 8 embryos show Gcm2 expression by three apparent primary parathyroids and a smaller accessory parathyroid (red arrow; 3/3 embryos; in each case the thymus/parathyroid primordium was examined from only one side of the embryo). The white arrow points to Gcm2 expressing cells that are still attached to the thymic domain. (K) higher magnification of the region boxed in J. pt, parathyroid; th, thymus; p3, 3^rd^ pharyngeal pouch; p4, 4th pharyngeal pouch.

To test whether a similar phenomenon occurs in human embryogenesis, we used whole-mount *in situ* hybridization for *Gcm2* in early week 6 to mid week 8 human embryos or dissected parathyroids and thymic lobes. At early week 6, *Gcm2* was expressed in the dorsal region of the 3^rd^ and 4^th^ pharyngeal pouches (Figure 4A, 4B; 2/2 embryos). By early week 7 the common parathyroid/thymic primordia (derived from the 3^rd^ pharyngeal pouch) have detached from the pharynx. Throughout week 7, clusters of *Gcm2* expressing cells were located in the anterior portion of the common primordium (4/4 embryos) and at the posterior tip (1/4 embryos) of the migrating elongated thymic structure (Figure 4C–4H). Similar to the phenomenon we found in mouse (Figure 2A-II), small clusters of ‘stray’ *GCM2-*positive cells were often present (Figure 4E, *). By late week 7, although *Gcm2* positive cells were still attached to the common primordia, separate parathyroids were present, as well as small *Gcm2* expressing clusters that may represent accessory parathyroids (Figure 4F–4H; 1/1 embryo).

Dissected parathyroids and thymic lobes from one side of early and mid week 8 embryos showed three major *Gcm2* expressing parathyroids (black arrowheads) and a smaller *Gcm2* expressing accessory parathyroid (red arrowhead) associated with a single thymic lobe (Figure 4I–4K; 3/3 embryos). Of the major parathyroid rudiments, one is clearly associated with the thymic primordium at late week 7 and therefore appears to derive from the 3^rd^ pharyngeal pouch, while the other is clearly outside the common thymus-parathyroid primordium and thus most likely derives from the 4^th^ pharyngeal pouch (note that *GCM2* expression is clearly evident in the 4^th^ pouch at week 6) (Figure 4I–4K). In addition, a smaller parathyroid rudiment was consistently observed associated with the posterior tip of the thymus domain of the common primordium at week 7 and week 8 (Figure 4D–4J), although at least in late week 7 this appeared to be present only in one of the two bilateral primordia (Figure 4H). Furthermore, as the parathyroid separates, some *Gcm2* expressing cells are left attached to the upper cordlike thymic structure (Figure 4J, 4K, white arrows). These data demonstrate that similar to our observations in the mouse, ectopic parathyroids exist from week 7 in the human embryo, and that the presence of intrathymic parathyroids in adulthood may be in part due to incomplete separation from the thymus.

### Thymic Epithelial Cells Express Thymic PTH Via a *Gcm2*-Independent Pathway

RT-PCR using total thymus cDNA from *Gcm2* ^−/−^ mice could still amplify *Pth* at high cycle numbers (Figure 3C), suggesting that the misplaced parathyroid cells were not the only source of thymic PTH. Quantitative RT-PCR using total thymus cDNA from wild-type and *Gcm2* ^−/−^ mice on a C57BL/6 genetic background showed that the second source of thymic PTH in the *Gcm2* ^−/−^ mice is about 1/350 of the level in the wild-type mice on the C57Bl/6 background (Figure 3D). We therefore investigated this Gcm2-independent source of thymic PTH expression.

**Figure 5:**
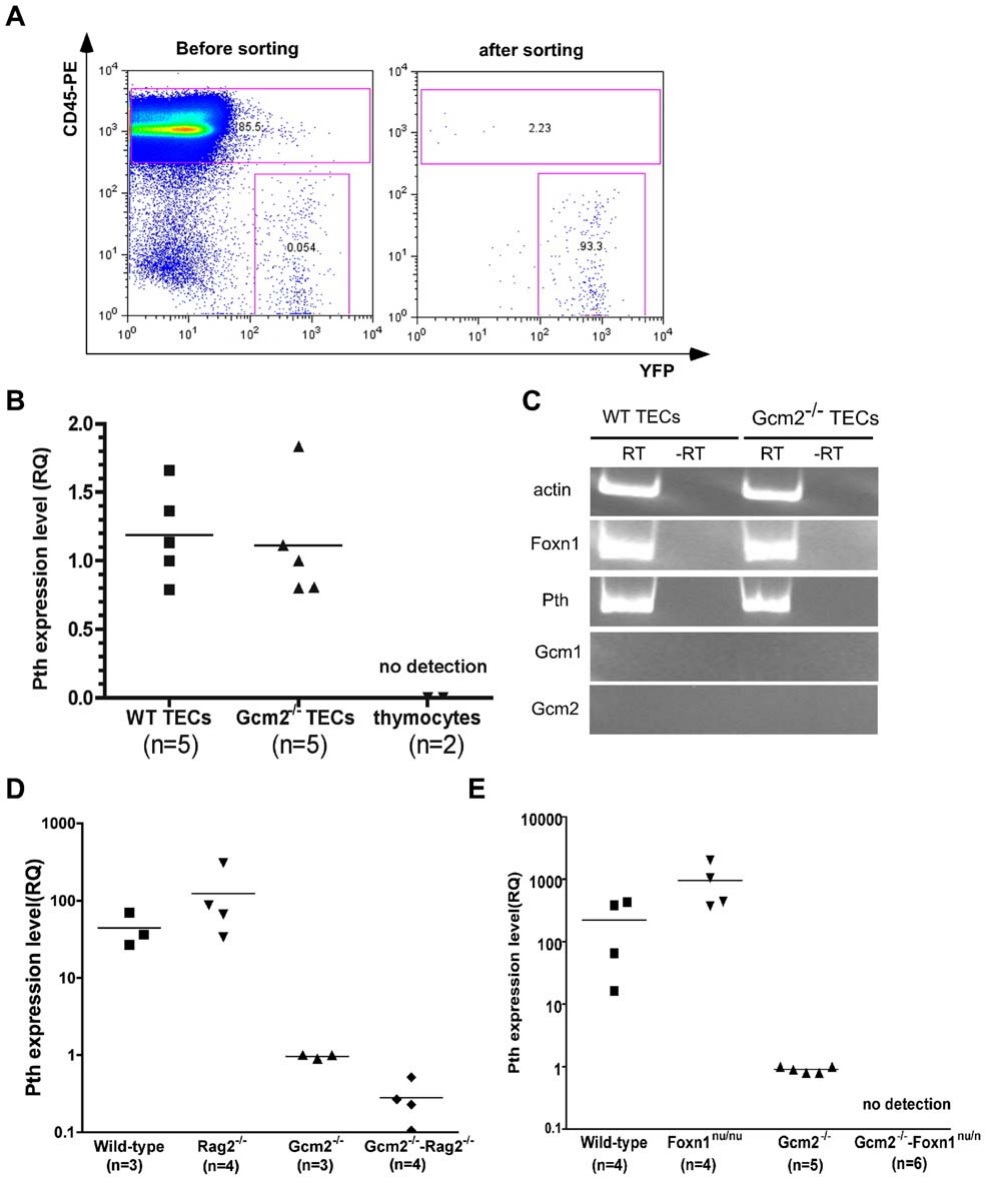
mTEC-derived PTH expression is Gcm2-independent but requires Foxn1-mediated TEC differentiation. (A) TEC sorting from control or *Gcm2* ^−/−^ adult thymi. TEC were labeled by activating expression from the R26YFP indicator using Foxn1Cre; cells were stained with anti CD45-PE to gate out CD45^+^ thymocytes. FACS analysis was used to check the purity after cell sorting. (B) Real-time PCR for *Pth* was performed on cDNA synthesized from sorted TECs from wild-type and *Gcm2* ^−/−^ adult thymi. Sorted CD45+ thymocytes were negative for *Pth*. (C) RT-PCR using cDNA synthesized from sorted TECs from wild-type (WT) or *Gcm2* ^−/−^ adult thymi. (D) Real-time PCR for *Pth* from total thymus cDNA samples from newborn wild-types (WT), *Rag2* ^−*/*−^, *Gcm2* ^−/−^, and *Gcm2* ^−/−^;*Rag2* ^−*/*−^ mutants. (E) Real-time PCR for *Pth* from total thymus from newborn wild-types, *Foxn1* ^nu/nu^, *Gcm2* ^−/−^, and *Gcm2* ^−/−^;*Foxn1* ^nu/nu^ mutants. RQ is the relative quantitative expression level of *Pth*. In panels B,D and E, n = number of thymi analyzed.

The thymus is a complex immune organ composed of hematopoietic cell-derived thymocytes and multiple types of stromal cells [22]. TECs play a required role in the production of a self-restricted and self-tolerant T-cell repertoire through positive selection and negative selection [22]. Negative selection occurs in the medullary region, where medullary TECs (mTECs) promiscuously express many tissue-restricted self-antigens (TRAs) that are required for negative selection to establish central tolerance and prevent autoimmunity [23]. To test whether thymic PTH expression was due to TRA expression in mTECs, we performed RT-PCR on sorted TECs (Figure 5). TECs expressed both *Foxn1* and *Pth*, and expression levels were similar in TECs sorted from wild-type controls and *Gcm2* ^−/−^ mutants (Figure 5B and 5C). We did not detect *Gcm1* or *Gcm2* expression in the purified TECs (Figure 5C), indicating that *Pth* expression in these cells is not controlled by *Gcm2*, and arguing against a previously proposed role for *Gcm1* in regulating thymic PTH expression [4].

*Pth* expression was not found in other thymic cell types by RT-PCR or microarray analyses, including T cells, macrophages, and dendritic cells (Figure 5B) [24]. Microarray data from sorted mTECs or cTECs also showed that *Pth* transcripts were present only in mTECs [24]. We further confirmed the expression of *Pth* in mTECs using Rag2^−/−^ mutant mice, which have a normal cortical structure but lack an organized medulla [25], [26]. Thymic *Pth* expression was greatly reduced in *Gcm2* ^−/−^;*Rag2* ^−/−^ double mutants (Figure 5D), although not totally ablated, consistent with the incomplete block in mTEC differentiation in Rag2 mutants.

### Analysis of *Gcm2;Foxn1* Double Mutants

As a genetic test of the TEC origin of thymic *Pth* expression, we generated *Gcm2* ^−*/*−^;*Foxn1^nu/nu^* double mutant mice that have no parathyroids and in which TEC differentiation is blocked [27]. We failed to detect any thymic *Pth* expression in the thymic epithelial rudiments of *Gcm2*;*Foxn1* double mutants (Figure 5E). These results further supported the conclusion that thymic *Pth* expression has only two sources: misplaced authentic parathyroid cells that express *Pth* in a *Gcm2*-dependent manner; and differentiated mTECs that express *Pth* independent of *Gcm2*.

**Figure 6:**
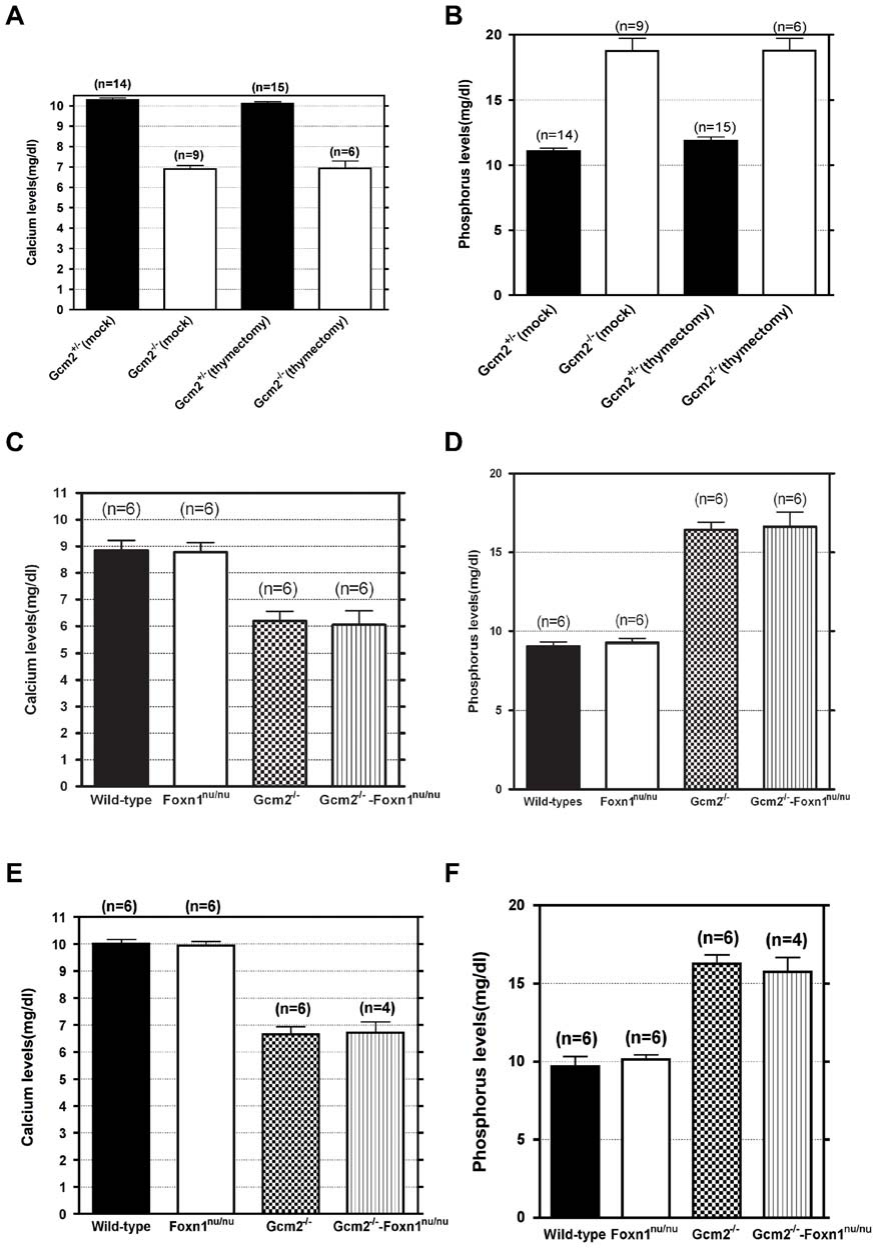
Neither thymectomy nor the *Foxn1^nu^* mutation exacerbated the hypoparathyroidism phenotype in *Gcm2* ^−/−^ mutants. (A–B) Ionized calcium and inorganic phosphorus concentrations of *Gcm2* ^−/−^ mutants and *Gcm2* ^+/−^ controls from mock and thymectomized groups. Thymectomy surgery did not alter ionized calcium and inorganic phosphorus concentration among mock and thymectomized groups with the same genotype (t test, P>0.05). Serum samples were collected from 1 month old adult mice. (C–D) Ionized calcium and inorganic phosphorus concentrations of newborn wild-type (WT), *Foxn1* ^nu/nu^ mutants, *Gcm2* ^−/−^ mutants and *Gcm2* ^−/−^ and *Foxn1* ^nu/nu^ double mutants. The serum samples were collected from newborn mice on the 129S6 and C57BL/6J F1 hybrid genetic background. (E–F) Ionized calcium and inorganic phosphorus concentrations of adult wild-type (WT), *Foxn1* ^nu/nu^ mutants, *Gcm2* ^−/−^ mutants and *Gcm2* ^−/−^ and *Foxn1* ^nu/nu^ double mutants. The serum samples were collected from 1 month-old adult mice on the 129S6 and C57BL/6J F1 hybrid genetic background. In panels C–F, Foxn1 null mutation did not alter ionized calcium and inorganic phosphorus concentration between groups with the same *Gcm2* genotype (t test, P>0.05). In all panels, n =  the number of mice analyzed.

The initial report of the *Gcm2* single mutant phenotype invoked the 100% neonatal lethality of *Hoxa3* mutants, which are aparathyroid and athymic, in support of the proposal that thymus-derived PTH ameliorated the lethality phenotype of *Gcm2* mutants [4]. As *Hoxa3* mutants have a variety of other defects that could contribute to lethality [28], [29], we used the *Gcm2* ^−*/*−^; *Foxn1^nu/nu^* double mutants as a more appropriate test of this possibility. These double mutants have a specific genetic deletion of both parathyroids and thymus, without any known potentially confounding phenotypes. In double heterozygote intercrosses, all genotypes were present in the expected Mendelian ratios at the newborn stage. Adult mice had reduced numbers of genotypes homozygous for the *Gcm2* mutation (Table 2), consistent with the rate of lethality of *Gcm2* ^−/−^ mutants on this mixed genetic background. Surprisingly, compared with a survival rate of about 55% for *Gcm2* ^−/−^ mutants in these crosses, *Gcm2* ^−*/*−^;*Foxn1^nu/nu^* double mutants had a lower survival rate of about 18% (Table 2). However, the ionized calcium and inorganic phosphorus concentrations in both newborn and adult mice were not significantly different between wild-type and *Foxn1^nu/nu^* mutant mice, or between *Gcm2* ^−/−^ mutants and *Gcm2*;*Foxn1* double mutants (Figure 6C–6F). These results indicate that Foxn1-dependent *Pth* expression in mTECs does not contribute to serum calcium physiology. While the reason for the increased lethality of double mutants is as yet unclear, these data provide further evidence that the lethality phenotype of *Gcm2* ^−/−^ and *Gcm2* ^−*/*−^ *Foxn1* ^−*/*−^ mutants was not PTH-related.

**Table 2:** Survival rates of *Gcm2* ^−/−^ single and *Gcm2* ^−/−^; *Foxn1^nu^* ^/*nu*^ double mutants on the 129S6/C57Bl6 hybrid background at weaning.

Genotype	Mendel Ratio	Observed number	Expected number	Survival rate	d^2^	d^2^/e
*Gcm2^+/+^; Foxn1^+/+^*	9/16	116	102.375		185.6	1.81
*Gcm2^+/+^; Foxn1^nu/+^*						
*Gcm2^+/−^; Foxn1^+/+^*						
*Gcm2^+/−^; Foxn1^nu/+^*						
*Gcm2^+/+^; * ***Foxn1^nu/nu^***	3/16	45	34.125		118.3	3.47
*Gcm2^+/−^; * ***Foxn1^nu/nu^***						
***Gcm2^−/−^*** *; Foxn1^+/+^*	3/16	19	34.125	55.7%	228.8	6.70
***Gcm2^−/−^*** *; Foxn1^nu/+^*						
***Gcm2^−/−^*** *; * ***Foxn1^nu/nu^***	1/16	2	11.375	17.6%	87.9	7.73
total	1	182	182			19.71

d =  deviation value, or subtraction of the expected from observed for each group.

e =  expected number.

chi-square value (Χ^2^)  = 19.71; P<0.001; degrees of freedom (df) = 3.

### mTEC-Derived PTH Does Not Have Endocrine Function in *Gcm2*-Null Mutant Mice

The initial report of the *Gcm2* null mutant phenotype showed that surgical removal of both the thymus and parathyroids from wild-type adults resulted in lethality [4]. As our data shows that parathyroid cells are normally associated with the thymus due to the incomplete organ separation during development, this result could have been due to the removal of thymus-associated parathyroids, rather than to the removal of thymus-produced PTH. Although there was no detectable serum PTH in most *Gcm2* mutants (Figure 1C), we tested whether thymic PTH participates in endocrine function by determining whether the removal of the thymus from *Gcm2* ^−/−^ mutants would increase lethality on the 129/C57BL/6J hybrid background. First, we performed thymectomy surgery on newborn *Gcm2* ^−/−^ mutants on the 129S-C57Bl/6 genetic background. Unmanipulated and mock surgery groups from the same 129S6/C57BL/6J hybrid genetic background were used as controls. Thymectomized *Gcm2* ^−/−^ mutants did not show increased lethality (Table 1), and serum biochemistry did not show any difference in ionized calcium or inorganic phosphorus levels between surviving *Gcm2* ^−/−^ mutants with mock surgery and *Gcm2* ^−/−^ mutants with thymectomy (Figure 6A, 6B). These data, in combination with the analysis of *Gcm2;Foxn1* double mutants, therefore demonstrate that the thymus does not provide any PTH-related endocrine function in mice.

## Discussion

Our data reveal two cellular sources of extra-parathyroid PTH. The first source is misplaced authentic parathyroid cells that arise during normal organogenesis, which express PTH in the same way as the parathyroid glands and are ablated in the *Gcm2* null mutants. The second source is mTECs, which express PTH independently of Gcm2, but dependent on Foxn1-mediated TEC differentiation. We also define two different physiological functions for the PTH derived from these two different sources. We propose that parathyroid cells, including those in the main parathyroid glands and the misplaced parathyroid cells, are the only physiologically relevant postnatal source of serum PTH, and that the thymus has no contribution to serum PTH or calcium physiology. mTECs also express PTH, probably as a self-antigen, but this PTH does not contribute to serum PTH for endocrine function. This result is consistent with the lack of secretory machinery in these cells used in the parathyroid cells to secrete PTH into the circulation [30]–[32], and the likelihood that the PTH translated in the mTECs is degraded into short peptides to be used for negative selection.

Based on our observations in both mouse and human, the separation process of the parathyroids from the thymus results in multiple “micro-parathyroids” in addition to the main parathyroid glands. Parathyroid adenomas have been found in the human thymus, and have been shown to express *Gcm2*, indicating that intrathymic adenomas could be the result of uncontrolled growth of the misplaced parathyroid cells [5], [6], [33]. These misplaced parathyroid cells could receive signals from the inappropriate microenviroment, causing them to secrete high PTH or over-proliferate; alternatively, these small groups of parathyroid cells may respond inappropriately to homeostatic mechanisms.

Our analysis presents the first genetic marker study of human parathyroid development, and reveals new information about parathyroid development that differs from the original descriptions of human parathyroid organogenesis based on histological studies. Our results on the ontogeny of these extra-parathyroid PTH-expressing cells provides insight into understanding the etiology of some hyperparathyroid disorders caused by ectopic parathyroid glands and intrathymic parathyroid adenomas [5], [6]. It is widely accepted that in humans four parathyroids develop during embryogenesis, giving rise to the superior and inferior parathyroids, and that ectopic and supernumerary parathyroids, often associated with the thymus, can cause primary hyperparathyroidism due to hyperplasia, adenomas, and carcinomas [5], [34]. Our data indicate that more than four major parathyroid rudiments are present by week 7 in the human fetus and that accessory parathyroids are present in the majority of fetuses at week 7 to week 8, and are therefore more frequent than previously documented [12], [35]. Our ability to identify these additional parathyroid structures is due to the increased resolution of analysis provided by *in situ* hybridization. Morphological studies would not identify all of the smaller accessory parathyroids, and may have annotated some parathyroid primordia as other structures.

In our view, it is not yet possible to definitively determine the relationship of the four GCM2-positive structures at week 8 to those present at late week 7, since the thymus and parathyroid primordia are actively migrating at this stage in development and lineage tracing studies are not possible in human embryos. The two GCM2-positive structures associated with the anterior end of the thymus primordium at week 8 may correspond to the major parathyroid foci present at late week 7 (i.e. to the structures we assign as arising from the 3^rd^ and 4^th^ pharyngeal pouches), while those at the posterior tip of the thymus primordium correspond to the Gcm2-positive clusters at the posterior in week 7. However, it is also possible that these both anterior parathyroids arise from the 3^rd^ pharyngeal pouch and that the 4^th^ pouch-derived parathyroid is no longer associated with the thymus primordium and therefore is not dissected out along with the thymus. In our view, the question of which of the parathyroid rudiments present in human fetal development give rise to the inferior and superior parathyroids in the adult also remains open, and will require further detailed study for its resolution.

Our original interest in this project was piqued by the differences in lethality between the *Gcm2* null mutants on different genetic backgrounds, from ∼30–60% on 129/C57BL/6J hybrid background (this report; [4]), to nearly 100% on the C57BL/6J background (this report). The original report of the *Gcm2* null mutants proposed that that thymic PTH could in part rescue an aparathyroid lethality phenotype; the lethality of thyroid-parathyroid-thymectomy in adult wild-type mice, and of athymic and aparathyroid *Hoxa3* null mutants were listed in support of this model [4]. It was later shown that *Pth* ^−/−^ mice can survive [7], [8], calling into question the assumption that aparathyroidism in mice would necessarily be lethal. Our data indicate that the lethality phenotype in *Gcm2* ^−/−^ mutants is not related to serum PTH levels; however, the question of why these mutants die is still not resolved. There are also unexplained differences in survival of different parathyroid-related mutants on the same background. A recent study from the Kovacs lab suggests that the measurable levels of PTH in some *Gcm2* ^−/−^ mutants at fetal stages may reflect Gcm2-independent PTH originating from the placenta. However, other than increased placental calcium transport, the *Gcm2* and *Pth* null mutants have very similar phenotypes, both of which are milder than the *Hoxa3* null when all are on the Black Swiss genetic background [19]. *Hoxa3* null mutants have phenotypes similar to the PTH/PTHrP null, which also die at birth, suggesting that Hoxa3 may play some role in calcium physiology outside the parathyroid. In part, the answer may lie outside of calcium physiology. *Hoxa3* mutants have other defects that could contribute to lethality [28], [29]. The increased postnatal lethality of the *Gcm2;Foxn1* double mutants may also be due to as yet unidentified functions for both of these transcription factors in other tissues; expression of both *Foxn1* and *Gcm2* has been identified in the postnatal central nervous system (http://mouse.brain-map.org/brain/Foxn1.html; http://www.ncbi.nlm.nih.gov/projects/gensat/).

Two models have been proposed for the mechanism that regulates the promiscuous expression of TRAs in mTECs. The progressive restriction model proposes a mosaic of TRA expression in immature mTECs characteristic of multi-lineage differentiated cells of endoderm-derived organs, and expressed by the same tissue-specific regulators as in their ‘normal’ tissues [36]. In contrast, the terminal differentiation model proposes that some mTECs have an autonomous property to express TRAs by a different mechanism compared to their tissue-specific regulation, characterized by lower transcriptional levels and independence from tissue-specific transcriptional regulators [24]. This latter model is supported by single-cell PCR of individual mTECs, and by the analysis of casein beta gene expression in mTECs compared to mammary gland cells [37]. Given the pharyngeal endodermal origin of parathyroid cells, PTH should be a good candidate for the progressive restriction model, as discussed above. However, our data showed a much lower *Pth* expression level in mTECs and a Foxn1-dependent and Gcm2-independent pathway for PTH expression in mTECs, more consistent with the terminal differentiation model; Gcm2-dependent PTH in the thymus came exclusively from misplaced parathyroid cells. Microarray analysis indicates that PTH expression in mTECs is Aire-independent [38], consistent with immunolocalization studies [39]. It is still an open question whether the regulation mechanism for thymic PTH in mTECs is common to other Aire-independent TRAs.

## Materials and Methods

### Ethics Statement

All experiments using mice were carried out at UGA with the approval of the UGA Institutional Animal Care and Use Committee. First and second trimester human fetuses were obtained in collaboration with the Reproductive Biology Unit, Little France, Edinburgh. Ethical approval for use of human fetal tissue was granted by the Lothian University Hospitals NHS Trust and the Lothian Research Ethics Committee: Smith et. al. ‘Isolation and propagation of fetal stem cells’ LREC/2002/6/15. Consent was obtained from all donors, and the tissue was anonymized before being made available for research. Use and disposal of tissues are strictly regulated in accordance with conditions stipulated in the Ethics approval and in the University of Edinburgh Health and Safety regulations regarding use of human tissue. All experiments using human tissue were performed at the University of Edinburgh.

### Mice

The generation and genotyping of *Gcm2* null mutant has been described [4]. *Gcm2* mutant mice on a 129/SvEv-C57BL/6J genetic background were backcrossed to C57BL/6J mice for more than 5 generations. These majority C57BL/6J *Gcm2* mutant mice were then backcrossed to 129S6 mice (Taconic) to obtain 129S6/C57BL/6 F1 hybrids.

*Foxn1-nude* mice (Jackson Labs) and R26YFP reporter mice [40] were maintained on a C57BL/6J and 129SvJ hybrid background. C57BL/6J Rag2 null mutant mice were a generous gift from Dr. E. V. Rothenberg. The Foxn1Cre allele of *Foxn1* was previously described [41].

Analysis of *Gcm2;Foxn1* double mutants was done by mating *Gcm2* ^+/−^; *Foxn1* ^+/*nu*^ males with *Gcm2* ^+/−^; *Foxn1* ^+/*nu*^ females. A total of 182 one month old mice from *Gcm2* ^+/−^; *Foxn1* ^+/*nu*^ mating were genotyped at weaning. Reduced survival of genotypes homozygous for the *Gcm2* mutation was significant using the chi-square test. Since there were no survival defects detected in *Gcm2* ^+/−^ or *Foxn1* ^+/*nu*^ heterozygous mice, we combined heterozygous mice with wild-type mice as a control group.

In all crosses, for calculating the % survival, the survival of wild-type mice was set at 100%. For staging of embryos, noon on the day of the vaginal plug was designated as E0.5.

### Human Fetal Tissue

Embryos were staged according to the standard head/rump measurement and classified according to Carnegie stages. Embryos used in this study were from week 6 (Carnegie stage 16–17), week 7 (Carnegie stage 18–19) and early to mid-week 8 (Carnegie stage 20–21). Embryos were fixed in 4% PFA for 24 hours and stored at −20°C in 100% methanol until used for analysis.

### RT-PCR and Semi-Quantitative RT-PCR

Isolation of RNA and RT-PCR were performed as described [42]. Tissues were dissected from embryos, newborns, or adult mice and total RNA was isolated with Trizol. Genomic DNA was removed using DNase I. Reverse transcription was performed using SuperScript III Reverse Transcriptase (Invitrogen), then cDNA was subjected to PCR. The following primers were used: β-actin forward 5′-TGGAATCCTGTGGCATCCATGAAAC-3′, β-actin reverse 5′-TAAAACGCAGCTCAGTAACAGTCCG-3′, *Pth* forward 5′-CTGCAGTCCAGTTCATCAGC-3′, *Pth* reverse 5′-AAGCTTGAAAAGGTAGCAGCA-3′, *Gcm2* forward 5′-CATCAATGACCCACAGATGC-3′, *Gcm2* reverse 5′-GGCACTTCTTCTGCCTTCTG-3′, *Foxn1* forward 5′-TGACGGAGCACTTCCCTTAC-3′, *Foxn1* reverse 5′-GGGAAAGGTGTGGGTAGGTC-3′, *Gcm1* forward 5′-TGAAAAACAAGCCCTTCAGC-3′ and *Gcm1* reverse 5′-TCTGGCTTTGTCACAGATGG-3′. Both *Gcm2* and *Pth* RT-PCR products were confirmed by sequencing.

### *In Situ* Hybridization

Paraffin section *in situ* hybridization for *Gcm2* and *Pth* was performed as described [3]. Staged embryos were fixed in 4% paraformaldehyde overnight and processed for paraffin embedding. 8-10 *µ*m sections were hybridized with digoxigenin-labeled RNA probes at 0.5 µg/ml. Alkaline phosphatase-conjugated antidigoxigenin Fab fragments were used at 1∶5000. BM-purple (Roche) was used as a chromagen to localize hybridized probe. Nuclear fast red was used as a counterstain.

Whole-mount *in situ* hybridization on human fetal tissue was performed as described [21]. Gcm2 probes used were generated by PCR amplification from microdissected human fetal thymic/parathyroid tissue using the following primers: Gcm2F, 5′-GGGCCACCTCCTATGAAAAT-3′; Gcm2R, 5′-GCAGCCTCTAGGGATGTGAA-3′. NBT/BCIP (Roche) was used to localize the hybidized probe. Embryos were embedded in paraffin and sectioned after staining in whole mount.

### Thymic Epithelial Cell Purification

Thymic stromal cell isolation was modified from a previously described method [43]. Thymi from *Foxn1^+/Cre^;R26-YFP^+/tg^* mice were dissected, minced into small pieces and agitated in RPMI1640 with 2% FBS to remove most thymocytes. The remaining tissue pieces were collected and resuspended in RPMI1640 and 0.2 mg/ml collagenase for 20 minutes at 37°C with gentle stirring. The tissue pieces were allowed to settle for 5 minutes, the supernatant was discarded, and the tissue was resuspended in dispase media (0.2 mg/ml of dispase, 0.2 mg/ml of collagenase and 25 ug/ml of DNaseI in RPMI 1640) for 20 minutes at 37°C with gentle stirring. The supernatant was discarded and the tissue chunks resuspended in fresh dispase media for 30–45 minutes at 37°C. The digested products were then passed through a 25 G needle, centrifuged at 800× g for 3minutes, resuspended in PBS containing 2% FBS and 5 mM EDTA, and then filtered through a 70 um cell strainer. The filtered cells were stained with anti-mouse CD45-PE (BD pharmingen) antibody before being subjected to sorting using a MoFlo cell sorter (Dako) to isolate PE^-^, YFP^+^ TECs. The yield of TECs was about 20,000 cells per adult thymus, with about 93% purity.

### RNA Preparation and Quantitative RT-PCR

Total RNA from sorted TECs was extracted with the RNeasy Micro kit according to manufacturer's instructions (QIAGEN). Total RNA from whole thymi was isolated with Trizol (Invitrogen). First-strand cDNA was reverse transcribed using superscript III (Invitrogen). Quantitative PCR was performed on an ABI 7500 real time PCR system with Taqman universal PCR mix (Applied Biosystems). 18S rRNA VIC/TAMRA primer-probe (Applied Biosystems) was used as endogenous control. *Pth* FAM primer-probe (Assay ID: Mm00451600_-_g1) was purchased from Applied Biosystems. PCR was performed at 50°C, 2 min; 95°C, 10 min; 40 cycles of 95°C for 15 sec; 60°C for 1 min. The relative quantity of gene expression was determined using 7500 SDS software (Applied Biosystems).

### Serum Biochemistry

Serum sample collection from E18.5 fetal, newborn or adult mice has been described [44], [45]. For fetal or newborn mice, the neck was incised to transect the carotid and jugular, and whole blood was collected into plain capillary tubes. For adult mice, blood samples were collected into capillary tubes from tail vein right after mice were sacrificed by cervical dislocation, or a cardiac puncture was used to obtain larger samples. Serum samples were prepared by centrifugation to remove blood cells, then stored at −20°C until assayed. The inorganic phosphorus and ionized calcium levels were measured using kits 117-30 (for phosphorus) and 140-20 (for calcium) from Diagnostic Chemicals Limited (Canada). Serum PTH was measured with a rodent PTH 1-34 Elisa kit with a detection limit of 1.6 pg/ml (Immutopics, San Clemente, CA). PTH values that were below the detection limit of 1.6 were reassigned a value equal to the detection limit.

### Thymectomy

Neonatal thymecotomy was performed as described [46]. Each newborn pup was chilled on ice for 1 minute, until unresponsive. A small incision was made in the center of the throat. The submandibular gland and muscle were moved aside with forceps, and the top portion of the sternum cut to expose the thymus. The thymus was removed with a kimwipe-covered toothpick, and the sternum and skin closed with surgical adhesive (3 M Vetbond, No. 1469SB, 3 M Animal Care Products, St. Paul, MN, USA). Pups were revived on a 37°C warming plate, then returned to their mother. Mock surgeries were performed without removing the thymus. All mice were allowed to grow until 1 month of age, then serum samples were prepared for serum biochemistry as described above.
